# Systematic prediction of key genes for ovarian cancer by co‐expression network analysis

**DOI:** 10.1111/jcmm.15271

**Published:** 2020-04-21

**Authors:** Mingyuan Wang, Jinjin Wang, Jinglan Liu, Lili Zhu, Heng Ma, Jiang Zou, Wei Wu, Kangkai Wang

**Affiliations:** ^1^ Department of Pathophysiology School of Basic Medical Science Central South University Changsha China; ^2^ Key Laboratory of Sepsis Translational Medicine of Hunan Central South University Changsha China; ^3^ Department of gynecology Zhuzhou Central Hospital Central South University Zhuzhou China; ^4^ Department of Geratic Surgery Xiangya Hospital Central South University Changsha China; ^5^ National Clinical Research Center for Geriatric Disorders Xiangya Hospital Central South University Changsha China; ^6^ Department of Laboratory Animals Hunan Key Laboratory of Animal Models for Human Diseases Xiangya School of Medicine Central South University Changsha China

**Keywords:** autophagy, ovarian cancer, prognosis, taxol (TAX) resistance, weighted gene co‐expression network analysis

## Abstract

Ovarian cancer (OC) is the most lethal gynaecological malignancy, characterized by high recurrence and mortality. However, the mechanisms of its pathogenesis remain largely unknown, hindering the investigation of the functional roles. This study sought to identify key hub genes that may serve as biomarkers correlated with prognosis. Here, we conduct an integrated analysis using the weighted gene co‐expression network analysis (WGCNA) to explore the clinically significant gene sets and identify candidate hub genes associated with OC clinical phenotypes. The gene expression profiles were obtained from the MERAV database. Validations of candidate hub genes were performed with RNASeqV2 data and the corresponding clinical information available from The Cancer Genome Atlas (TCGA) database. In addition, we examined the candidate genes in ovarian cancer cells. Totally, 19 modules were identified and 26 hub genes were extracted from the most significant module (*R* ^2^ = .53) in clinical stages. Through the validation of TCGA data, we found that five hub genes (COL1A1, DCN, LUM, POSTN and THBS2) predicted poor prognosis. Receiver operating characteristic (ROC) curves demonstrated that these five genes exhibited diagnostic efficiency for early‐stage and advanced‐stage cancer. The protein expression of these five genes in tumour tissues was significantly higher than that in normal tissues. Besides, the expression of COL1A1 was associated with the TAX resistance of tumours and could be affected by the autophagy level in OC cell line. In conclusion, our findings identified five genes could serve as biomarkers related to the prognosis of OC and may be helpful for revealing pathogenic mechanism and developing further research.

## 1 INTRODUCTION

Ovarian cancer (OC), the most fatal gynaecological malignancy, is characterized by early diagnosis difficulty, rapid metastasis and high recurrence rate.^1^ Despite good therapeutic response in early stage, most patients are diagnosed in advanced stage with poor overall survival.^2^ These features are related to the biological mechanism, which is the key determinant of outcome.^3^ Current therapeutic strategies of OC have improved significantly. The outcome depend on numbers of factors, including the patient's age, physical condition and stage at presentation.^4^ However, the 5‐year survival rate for advanced OC is much lower than in early stage, and about 70% of patients relapse.^5^

Previous study has linked OC recurrence to histological type, FIGO stage and chemotherapy regimens.^6^ Numerous genetic variations occur during the development of the tumour. With the help of large‐scale screening and bioinformatics, hundreds of genes alterations have been revealed to be closely related to the development and prognosis of tumours.^7^ However, studies of hub genes remain lacking. Therefore, more meaningful biomarkers need to be explored.

The weighted gene co‐expression network analysis (WGCNA) is a powerful technique developed by Langfelder and Horvath.^8^ It is widely used as a systematic biology method to describe the correlation patterns between genes and clinical features. Thus, in the present study, we focused on the association between the gene sets and common OC phenotypes and identified novel biomarkers for better OC prognostic investigation.

In many cases, recurrence leads to adverse reactions to chemotherapy.^9^ And tumour recurrence is closely related to drug resistance. In fact, studies have exhibited that drug‐resistant cancer cells augmented activation of autophagy, which seems to be a major obstacle in chemotherapy.^10^, ^11^ In this context, we validated the candidate hub gene COL1A1 which was regulated by autophagy and affected the drug sensitivity of tumour cells. This indicates that our screening candidates demonstrate the prognostic value of OC.

## 2 MATERIALS AND METHODS

### 2.1 Data procession

The gene expression profiles of OC were extracted from the MERAV (MERAV, http://merav.wi.mit.edu) database. Data adjustments included data filtering, log2 transformation and normalization. All data were summarized using ‘Affy’ package from Bioconductor (http://www.bioconductor.org/). Then, the top 25% most variant genes were selected for subsequent WGCNA.

### 2.2 Establishment of weighted co‐expression network

The chosen variant genes were constructed to an approximate scale‐free fundamental gene co‐expression network using the R package ‘WGCNA’.^8^

In order to calculate the connection strength between each pair of genes, the adjacency matrix *a_ij_* was defined as follows:

$$s_{\mathrm{ij}}=\left|\text{cor}(x_{i},x_{j})\right|a_{\mathrm{ij}}=S_{\mathrm{ij}}^{\beta }$$

*a_ij_* encoded the adjacent network connection strength between gene *i* and gene *j*, *x_i_* and *x_j_* were vectors of expression value for gene *i* and *j*, and *s_ij_* represented Pearson's correlation coefficient between gene *i* and gene *j*. For network generation, genes with a high correlation coefficient were clustered. The network modules were generated using the topological overlap measure (TOM),^12^ which was calculated using the adjacency matrix.

$${\text{TOM}}_{i,j}=\frac{\Sigma_{K=1}^{N}\;A_{i,k}\cdot \;A_{k,j}+A_{i,j}}{\min\left(K_{i},K_{j}\right)+1-A_{i,j}}$$

The adjacency matrix *A_ij_* was defined as follows:

$$A_{\mathrm{ij}}={\left|\text{cor}(x_{i},x_{j})\right|}^{\beta }$$

According to the TOM‐based dissimilarity measure, the network was built with the function blockwiseModules, where the minimum module size (minModuleSize) was 30. Average linkage hierarchical clustering was conducted to classify genes with high correlation coefficients into the same modules. DynamicTreeCut algorithm was used to identify the co‐expression module, where MergeCutHeight = 0.25 merges modules with a similarity of 0.75.

### 2.3 Clinical significant relevant modules identify

After obtaining the gene modules, we combined the clinical information with the genes in modules to analyse gene significance (GS) and the modular membership (MM), which were used to measure the correlation between the sample traits and the gene modules. Next, the targeted module genes were visualized with Cytoscape 3.6.1 software.^13^

### 2.4 Functional enrichment annotation

The targeted gene module was annotated, visualized and analysed using g:Profiler (https://biit.cs.ut.ee/gprofiler/gconvert.cgi) with default settings. The enrichment contains biological process (BP), molecular function (MF), cellular component (CC) and Kyoto Encyclopedia of Genes and Genomes (KEGG). The cut‐off criterion was *P* < .001. The results were demonstrated in the form of networks.

### 2.5 Hub gene identification and validation

Hub genes are the genes that rank high in connectivity in a module. They are located at the central hub, which can represent the characteristics of the module. Compared with all genes in the network, the hub genes in the module have greater biological significance. In this study, the protein‐protein interaction (PPI) information was extracted from the STRING database (http://string‐db.org/) and visualized using Cytoscape software. Subsequently, the plug‐in molecular complex detection (MCODE) of Cytoscape was used to construct the subnetwork for further validation.

Kaplan‐Meier plotter (www. kmplot.com) was used to perform progression‐free survival (PFS) analyses of hub genes.^14^ RNA sequencing data and the corresponding clinical information of OC were extracted from The Cancer Genome Atlas Project (TCGA, https://cancergenome.nih.gov/) database and normalized using edgeR package. The expression of hub genes was measured by the immunohistochemistry using the Human Protein Atlas (http://www.proteinatlas. org).

### 2.6 Cell culture

The human ovarian carcinoma A2780 cell line was purchased from ATCC (American Type Culture Collection), and TAX was obtained from Sigma (Poole, UK). TAX‐resistant A2780R cell line was developed by exposing them to cyclic TAX treatment. Cells were cultured in Dulbecco's modified Eagle's medium (DMEM) containing 10% foetal bovine serum or serum‐free DMEM at 37°C with 5% CO_2_ and incubated for the following experiments. 3‐methyladenine (3‐MA), dissolved directly in media, was purchased from Selleck Chemicals, and cells were treated with 3‐MA at the concentration of 10 mmol/L for 24 hours.

### 2.7 CCK‐8 assay

Cell viability was evaluated by performing a Cell Counting Kit‐8 (Dojindo) assay and was measured with the BioTek Gen5 system (BioTek) at OD 450 nm.

### 2.8 Western blot analysis

Cells in different groups were collected, respectively, and were lysed in radioimmunoprecipitation assay (RIPA) lysis buffer (Beyotime Biotechnology) containing 1% protease inhibitor cocktail. Protein quantitative processing was performed with the BCA Kit (Sigma). Protein samples were separated on 10% SDS‐polyacrylamide gel and blotted to polyvinylidene difluoride (PVDF) membranes. Immunoblotting was incubated with the primary antibody (1:1,000 dilutions) at 4°C overnight and the secondary antibody at room temperature for 2 hours. Immunostaining was performed with ECL Western blotting detection reagent (Thermo).

### 2.9 RNA extraction and qRT‐PCR

For qRT‐PCR analyses, total RNA was extracted using an RNA extraction kit (Takara Bio) according to the manufacturer's instructions. cDNA syntheses were performed with PrimeScript RT Master Mix (Takara Bio) and amplified with SYBR pre‐mix EX Taq (Takara Bio). The qRT‐PCR analyses were performed on a 7500 PCR system (Applied Biosystems) with the primers shown in Table S1.

### 2.10 Statistical analysis

The sensitivity and specificity were depicted by receiver operating characteristic (ROC) curves using GraphPad Prism software. The log‐rank test was used to compare survival curves. Student's *t* test was used to compare the differences between groups, and *P* < .05 was considered statistically significant. Statistical analyses were conducted using R software (version 3.5.0).

## 3 RESULTS

### 3.1 Construction of weighted co‐expression network and identification of key modules

**FIGURE 1:**
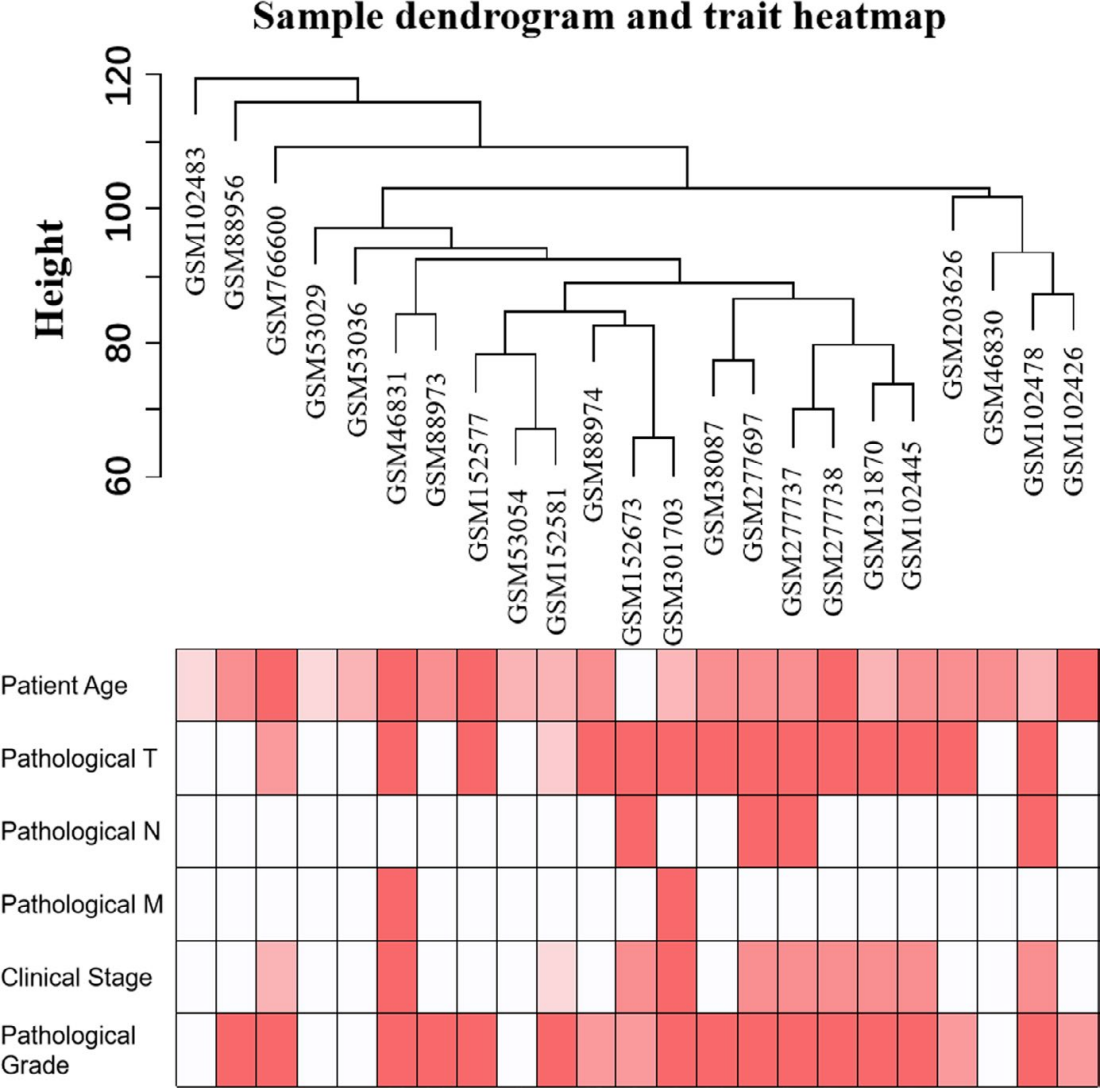
Clustering dendrogram of 23 samples

**FIGURE 2:**
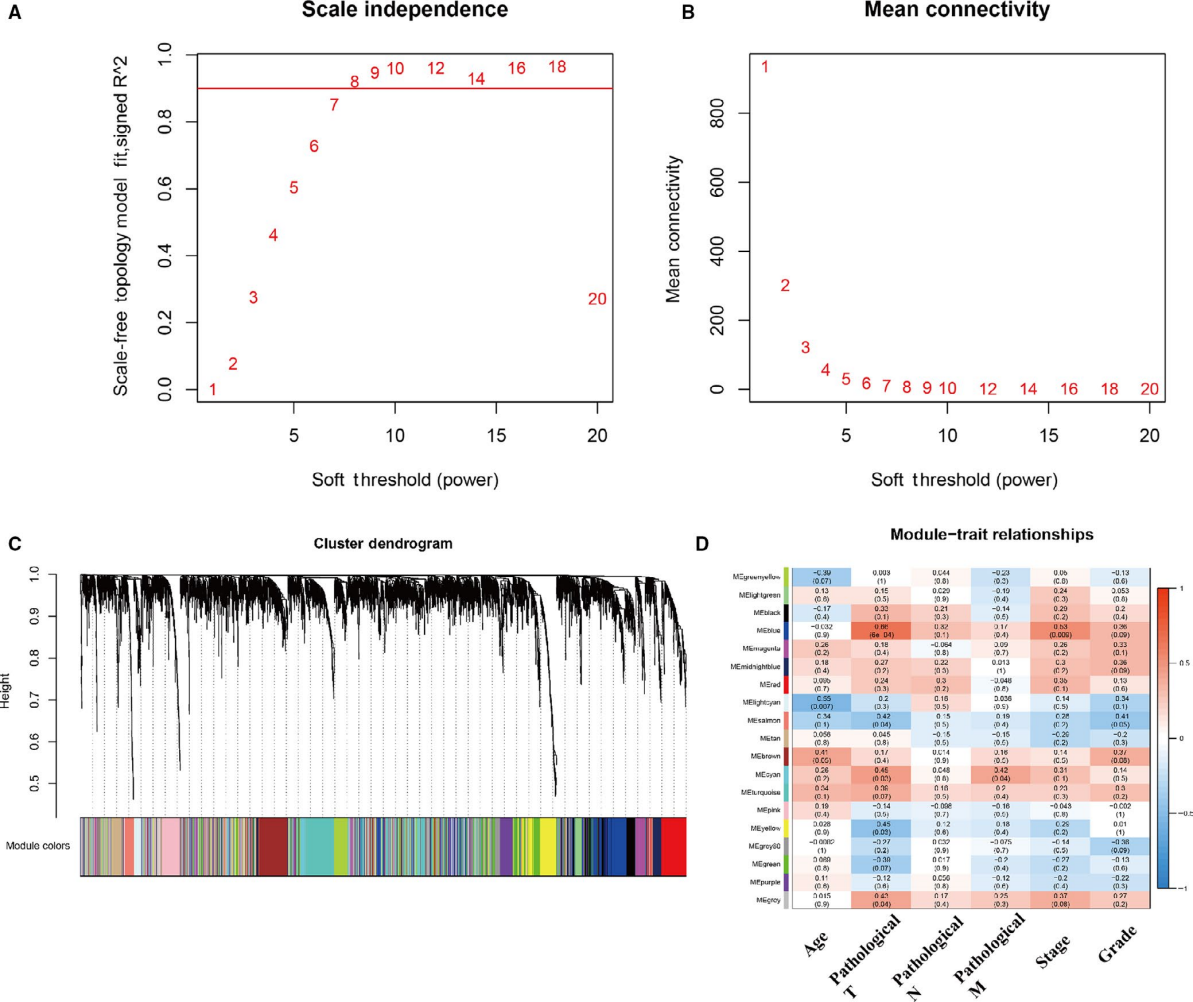
Determination of soft‐thresholding power in the WGCNA. A, Analysis of the scale‐free fit index for various soft‐thresholding powers (β). B, Analysis of the mean connectivity for various soft‐thresholding powers (β). We choose the lowest β that results in approximate scale‐free topology. Identification of modules associated with the clinical traits of ovarian cancer. C, Dendrogram of all differentially expressed genes clustered based on a dissimilarity measure (1‐TOM). The colour band provides a simple visual comparison of module assignments. The colour band shows the results from the automatic single block analysis. D, Heat map of the correlation between module eigengenes and clinical traits of ovarian cancer

After data pre‐processing, the top 25% most variant genes were selected for subsequent analyses. The samples were clustered using average linkage method and Pearson's correlation analysis (Figure 1). In this study, to ensure a scale‐free network, the power of *β* = 8 (*R* ^2^ = .919) was chosen for the soft‐threshold parameter (Figure 2A‐B). Nineteen modules were identified based on the average linkage hierarchical clustering (Figure 2C). The relationships between the modules and clinical traits were analysed. Finally, the blue module was found significantly correlated with clinical stages and pathological T factor (*P* < .01, Figure 2D). Total genes of the blue module are shown in Table S2.

### 3.2 Functional enrichment analysis and protein‐protein network construction

**FIGURE 3:**
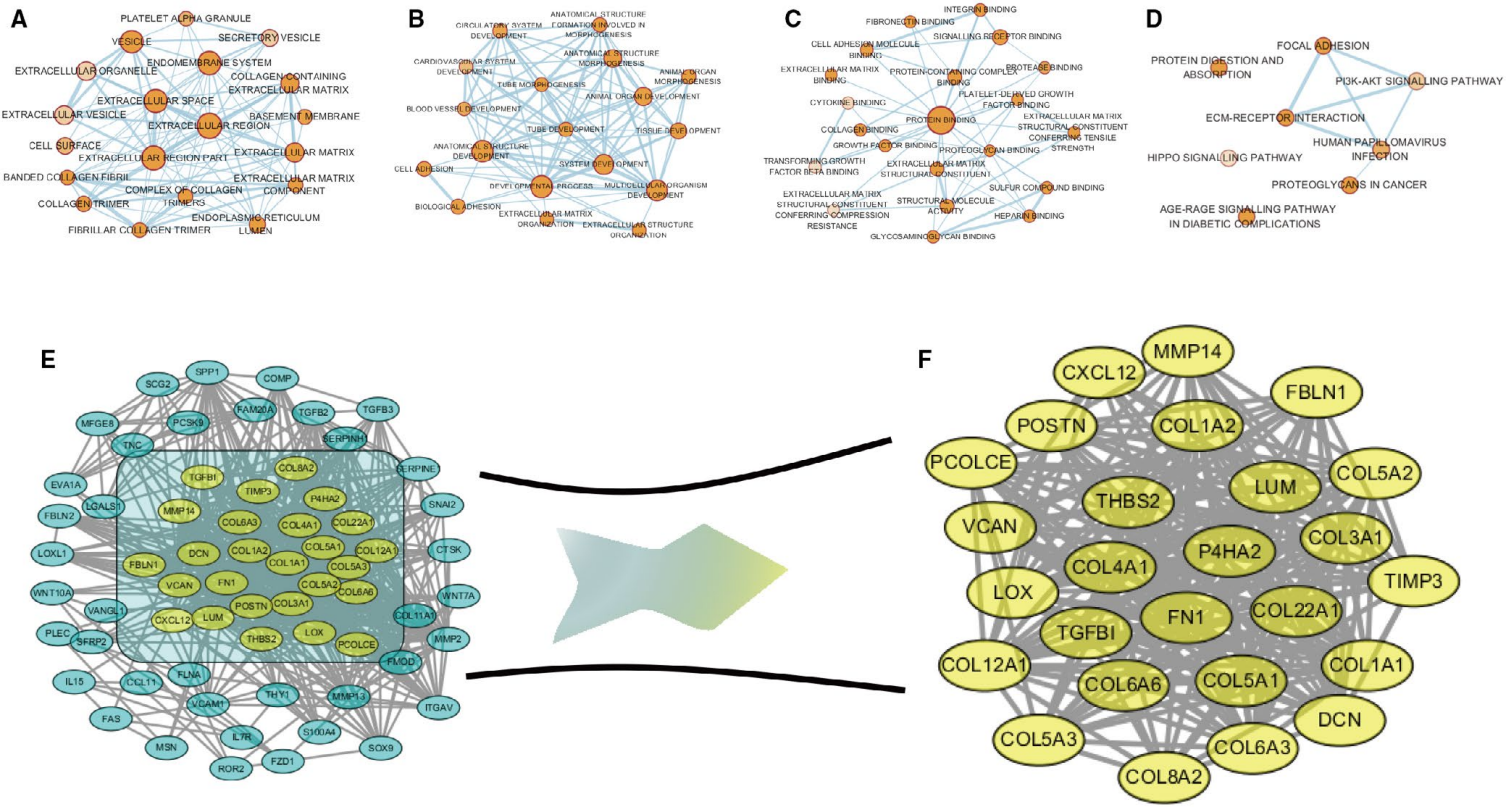
Gene ontology and pathway enrichment and network analysis of blue module genes. Biological process analysis (A). Cellular component analysis (B). Molecular function analysis (C). KEGG pathway analysis (D). Protein‐protein network of blue module genes (E). Subnetwork, the yellow nodes represent hub genes in the module (F)

The genes in blue module were categorized into four groups, including biological process (BP), cellular component (CC), molecular function (MF) and Kyoto Encyclopedia of Genes and Genomes (KEGG) pathway analysis. In the BP group, genes were mainly enriched in extracellular region, endomembrane system and vesicle; in the CC group, anatomical structure development, multicellular organism development and development process; and in the MF group, protein binding, growth factor binding and structural molecule activity. According to KEGG pathway analysis, genes were mainly involved in ECM‐receptor interaction, PI3K‐AKT signalling pathway and protein digestion and absorption (Figure 3A‐D).

Based on the protein associations obtained from the String database and the utilization of MCODE algorithm, 26 highly interconnected genes were selected to better characterize the blue module and were identified as hub genes (Figure 3E‐F).

### 3.3 Validation of hub genes

**FIGURE 4:**
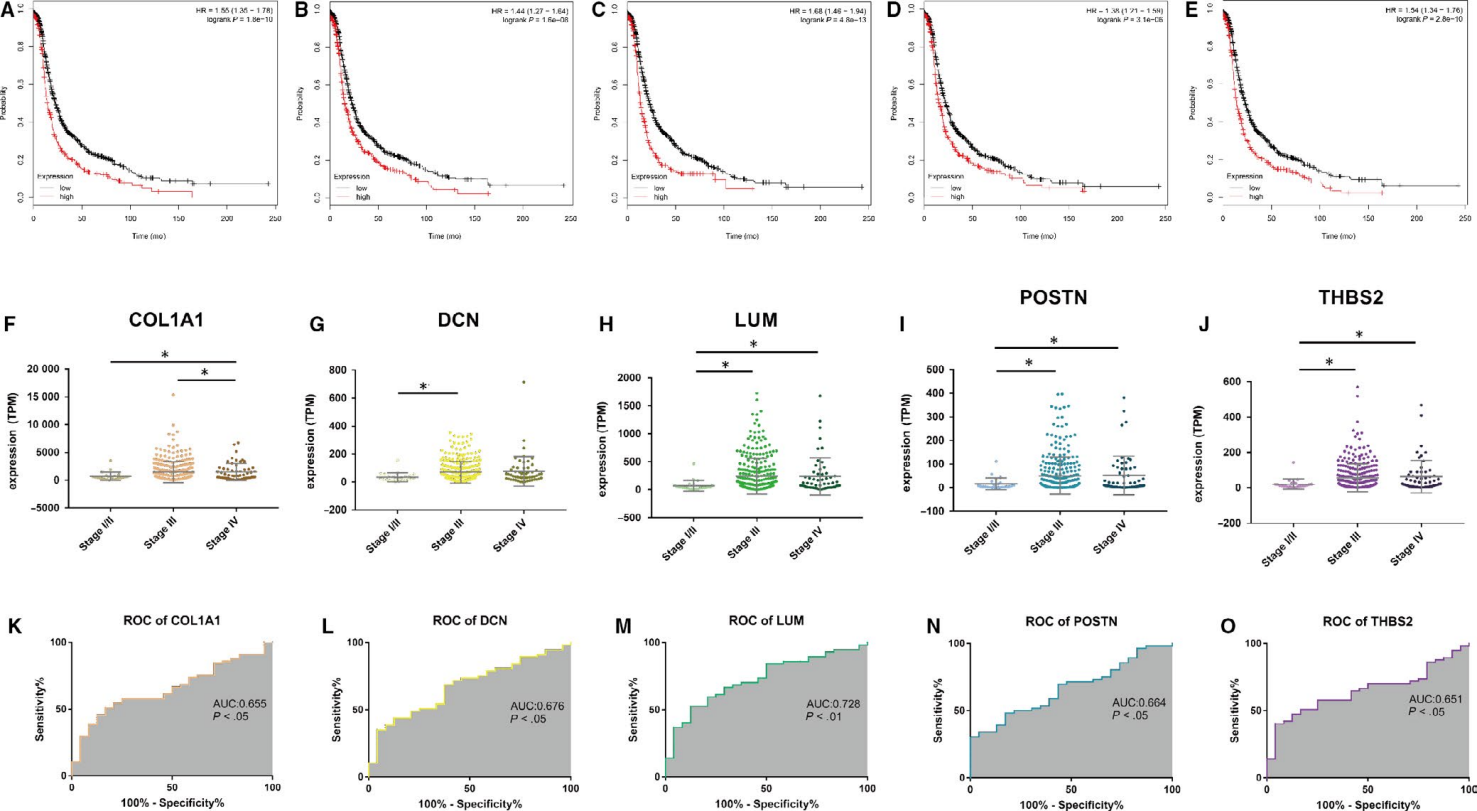
Progression‐free survival (PFS) of the five hub genes in ovarian cancer based on Kaplan‐Meier plotter. The patients were stratified into high‐level group and low‐level group according to median expression. (A) COL1A1. (B) DCN. (C) LUM. (D) POSTN. (E) THBS2. The correlation of gene expression of COL1A1, DCN, LUM, POSTN and THBS2 with clinical stages. The mRNA levels of COL1A1 (F), DCN (G), LUM (H), POSTN (I) and THBS2 (J). *P < .05. One‐way analysis of variance (ANOVA) was used to evaluate the statistical significance of differences. Receiver operator characteristic (ROC) curve analysis, early stage (I/II) vs advanced stage (III/IV). (K) COL1A1, (L) DCN, (M) LUM, (N) POSTN and (O) THBS2

**FIGURE 5:**
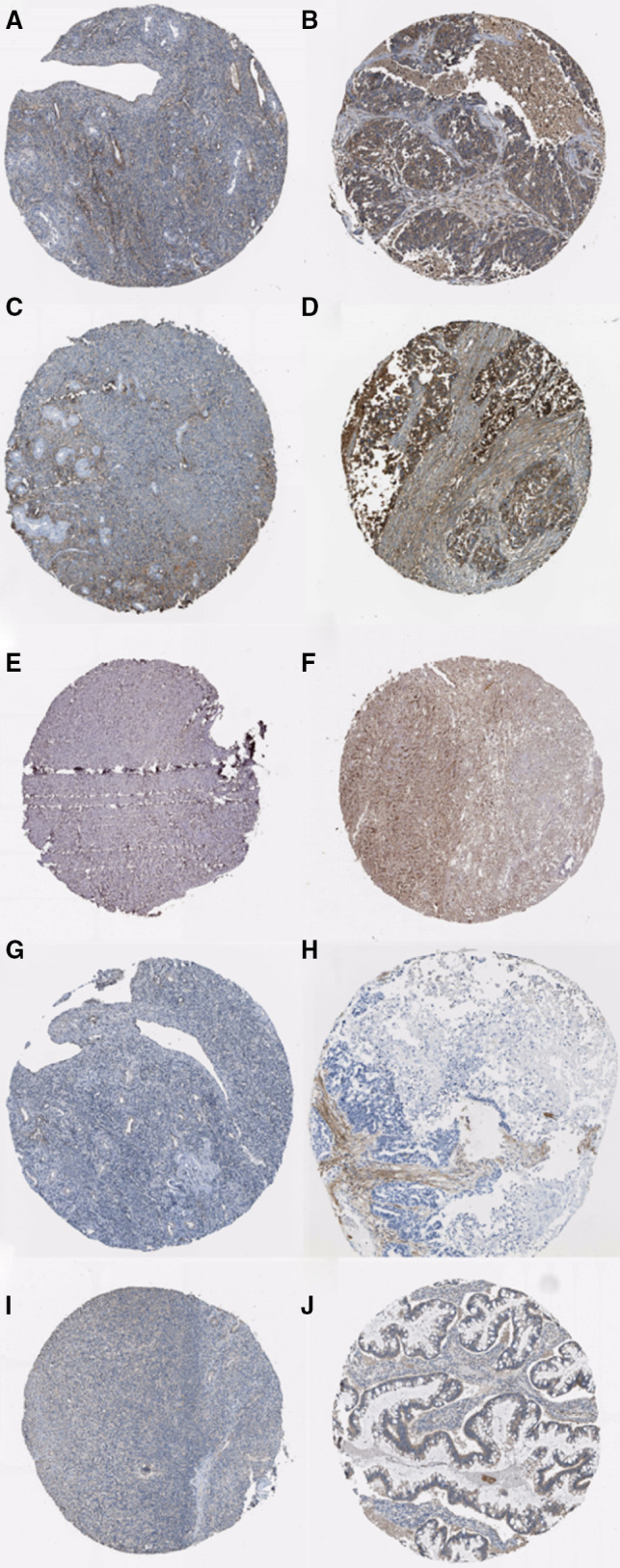
Immunohistochemistry of the five hub genes based on the Human Protein Atlas. A, Protein levels of COL1A1 in normal tissue (staining: high; intensity: moderate; quantity: <25%). B, Protein levels of COL1A1 in tumour tissue (staining: high; intensity: strong; quantity: >75%). C, Protein levels of DCN in normal tissue (staining: low; intensity: moderate; quantity: <25%). D, Protein levels of DCN in tumour tissue (staining: high; intensity: strong; quantity: >75%). E, Protein levels of LUM in normal tissue (staining: low; intensity: moderate; quantity: <25%). F, Protein levels of LUM in tumour tissue (staining: high; intensity: strong; quantity: >75%). G, Protein levels of POSTN in normal tissue (staining: not detected; intensity: weak; quantity: <25%). H, Protein levels of POSTN in tumour tissue (staining: medium; intensity: moderate; quantity:<25%). I, Protein levels of THBS2 in normal tissue (staining: medium; intensity: strong; quantity: <25%). J, Protein levels of THBS2 in tumour tissue (staining: medium; intensity: strong; quantity: 25%‐50%)

To further validate the prognostic value of the hub genes, we conducted survival analyses of the hub genes. Then, five genes including COL1A1, DCN, LUM, POSTN and THBS2 were found negatively associated with the PFS (Figure 4A‐E). In order to verify these five genes, expression levels of the selected genes were evaluated based on the TCGA data. In 379 OC samples from TCGA, the expression levels of these five genes were obviously up‐regulated in advanced tumour stages (Figure 4F‐J). ROC curve analyses showed that COL1A1, DCN, LUM, POSTN and THBS2 exhibited diagnostic efficiency for early‐stage and advanced‐stage OC (Figure 4K‐O). In addition, immunohistochemistry data from the Human Protein Atlas demonstrated that protein levels were higher in tumours compared with normal samples (Figure 5A‐J).

### 3.4 Construction of tax‐resistant cells and experimental validation of COL1A1

**FIGURE 6:**
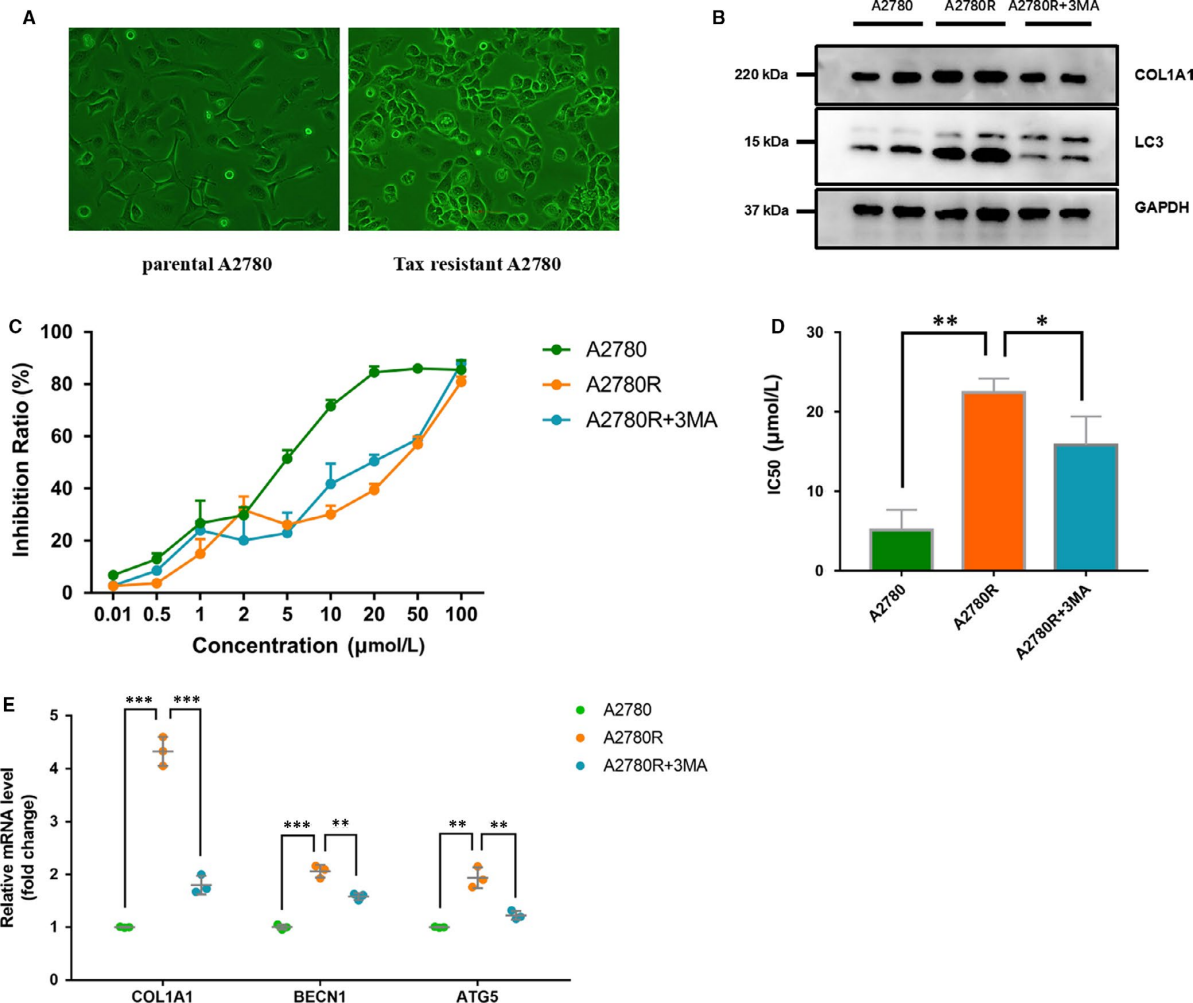
Experimental validation of COL1A1. A, Cellular morphological difference between parental A2780 and Tax‐resistant A2780R cell line. B, Western blotting showing the expression levels of COL1A1, LC3 and GAPDH. C, D, Inhibition rate of different TAX concentrations on A2780, A2780R and A2780R + 3MA, respectively. The difference in TAX resistance represented by IC50 (IC50, 5.109± 1.478 μmol/L vs 15.82± 2.07 μmol/L vs 22.42± 1.007 μmol/L). E, Quantitative real‐time PCR (qRT‐PCR) was performed to analyse the mRNA levels of COL1A1 and autophagy (BECN1 and ATG5) genes (n = 3). *P < .05; **P < .01; and ***P < .001. Student's *t* tests were used to evaluate the statistical significance of differences. BECN1: Beclin 1; ATG5: autophagy‐related 5

To further test the importance of the hub genes, COL1A1 was selected as we also previously reported.^15^ Drug resistance of OC chemotherapy is an important factor affecting prognosis, and TAX is currently the first line of chemotherapy. Studies have shown that the autophagy level of drug‐resistant tumour cells was higher than that of parental tumour cells.^16^, ^17^ Therefore, we constructed the A2780 TAX‐resistant OC cell line and verified the expression change in COL1A1 gene and the autophagy level. Tax‐resistant cells showed considerable difference in cell morphology (Figure 6A). After adding the autophagy inhibitor (3‐MA) to the A2780R cells, COL1A1 expression was significantly lower compared with GAPDH, indicating that COL1A1 was affected by autophagy in TAX‐resistant OC cells (Figure 6B). In order to explore the relationship between autophagy and TAX resistance, we adopted CCK‐8 method to detect cell activity at different TAX concentrations. The difference in TAX resistance among groups was then determined by half‐maximal inhibitory concentration (IC50). In A2780R group, IC50 was significantly higher than the parental A2780 group (22.42 ± 1.007 μmol/L vs 5.109 ± 1.478 μmol/L, *P* < .05, Figure 6C‐D), and 3‐MA reduced drug resistance compared with A2780R group (IC50, 15.82 ± 2.07 μmol/L vs 22.42 ± 1.007 μmol/L, *P* < .05, Figure 6C‐D). This suggested that autophagy could affect TAX resistance in OC cells. Further, we found that the mRNA expression level of COL1A1 was related to TAX resistance of OC cells. With the inhibition of autophagy, COL1A1 mRNA expression showed a similar trend (Figure 6E). This indicates that COL1A1 is related to the autophagy level and TAX resistance of OC cells. And the prognostic value was further demonstrated.

## 4 DISCUSSION

Ovarian cancer (OC), one of the three common gynaecological malignancies, ranks seventh among the tumours in women.^18^ There are numerous factors that affect the prognosis of OC, and the mechanism is complicated. Most OC patients will undergo clinical standardized treatment, but still develop tumour recurrence in 6‐18 months after treatment; however, advanced OC has a worse prognosis.^19^  Therefore, the analyses of OC’s clinical stages and drug‐resistance have important references value. It is necessary to conduct in‐depth analyses in clinical and basic researches to find out the biomarkers for the prognosis and explore their mechanisms.

In the current exploratory research, it is very important to construct a gene co‐expression network, which can help us identify genes related to diseases.^20^ Gene sets of weak effect are difficult for traditional analysis, but the WGCNA system is a good supplement, and modules can integrate weakly affecting genes. WGCNA has been successfully applied in the study of disease pathogenesis, classification, diagnosis and prognosis. After the power function processing, WGCNA will not make strong correlation relationships affected; however, the weak correlation relationships decrease significantly, which leads to the unsigned relationship network. Comparing with the conventional clustering method, the non‐scale network greatly demonstrates the whole physiological process of genes involved in the biological process, and the results are more credible. In this study, WGCNA was used to analyse the gene expression data of OC and 19 independent modules were obtained, among which blue module was the most relevant to OC clinical stage. Finally, we identified five genes (COL1A1, DCN, LUM, POSTN and THBS2) that are associated with clinical phenotype and may serve as potential new biomarkers.

Autophagy plays a complex role in human cancer, which is influenced by tumour micro‐environment, carcinogenic mutation type and other factors. It can effectively inhibit tumour growth in early stage. However, when cancer has suffered a long‐term stimulation, autophagy could degrade lipids and proteins to produce ATP, which promotes the development and growth of cancer.^17^ Among the five candidate biomarkers, the collagen type I alpha 1 chain (COL1A1) was found to be closely related to the development of OC, which was consistent with our previous study.^15^ Therefore, we constructed the A2780 TAX resistance cell line and attempted to verify the relationships among COL1A1, autophagy and TAX resistance of OC. The results showed that the TAX resistance of tumour cells was positively correlated with the autophagy level. Meanwhile, the inhibition of autophagy also decreased the expression of COL1A1. This indicates that COL1A1 is closely related to chemotherapy resistance and clinical stages, which demonstrated its prognostic value.

Collagen, the primary component of extracellular matrix (ECM), is the most abundant protein in the body. It ensures the structural integrity of tissues and organs and is closely related to the early development of the human body, cell‐cell connection, organ formation, platelet aggregation, cell chemotaxis, membrane permeability and other functions.^21^ The entire family of collagen, encoded by more than 30 different genes, contain 19 types of collagen.^22^ Type I collagen (COL1) is found in different connective tissues of the human body and is most abundant in human tissues. COL1 consists of two alpha 1(I) chains (COL1A1) and one alpha 2(I) chain (COL1A2) in a triple helix structure. The three peptide chains intertwine with each other to form a long helix, forming the unique triple‐helix structure of the collagen molecule. In OC, the degradation of mature COL1 reflects the clinical changes in cytotoxic chemotherapy and indicates the prognosis.^23^ Our results indicated that the expression of COL1A1 was increased in OC tissues compared with normal tissues and that its expression was significantly associated with clinical stages and TAX resistance.

Decorin (DCN), an important component of ECM, belongs to the small leucine‐rich proteoglycans (SLRPs) family. DCN is a three‐dimensional structure with four different domains, which can interact with a variety of cytokines or membrane receptors and participate in the regulation of collagen fibre formation.^24^, ^25^ Studies have found that DCN can inhibit the proliferation and migration of a variety of tumour cells in vitro, such as liver cancer, kidney cancer and breast cancer.^26^, ^27^, ^28^ In this study, it was found that DCN expression in tumour tissues was significantly higher than that in normal tissues, but the difference in advanced and early OC was not significant, suggesting that DCN is an oncogene in tumorigenesis, but it has no predictive effect on tumour development.

Lumican (LUM), an important component of ECM, is a mainly keratin‐rich polymeric protein originally found in the corneal stroma and is also a member of the SLRPs‐related family. It is widely expressed in human skin, blood vessels, lungs, breast, pancreas, colorectal, articular cartilage and other tissues.^29^ Aberrant expression of LUM can further affect the metastasis and invasion of tumour.^30^ In breast cancer, high expression of LUM indicates poor prognosis.^31^ However, LUM can induce proteoglycan composition changes and regulate the cell cycle to participate in tumour development.^32^ In the validation data of TCGA, our results indicated that LUM was significantly up‐regulated in OC tissues, and even higher in advanced stage than in early stage.

Periostin (POSTN) is a cellular adhesive protein. As an ECM protein, POSTN has been found to be highly expressed in a variety of malignancies and is often associated with recurrence, metastasis and poor prognosis.^33^, ^34^, ^35^ In OC, study has found that POSTN expression was substantially higher with chemotherapy resistance specimens than in those with chemotherapy‐sensitive patients.^36^ In our analysis, POSTN expression is negatively correlated with PFS, and the expression is more significantly up‐regulated in advanced OC.

As an important member of the thrombospondins (THBS) family, thrombospondin‐2 (THBS2) participates in a variety of cellular biological processes by binding ECM proteins and cell‐surface receptors.^37^ Studies have shown that THBS2 plays an important role in tumours and is related to the degree of malignancy.^38^, ^39^, ^40^, ^41^, ^42^ In this study, we found that THBS2 may be related to the clinical stage of OC and may be used as a biomarker for the prognosis.

All five biomarkers we screened are closely related to the formation of ECM. This may shed light on the pathophysiological mechanism of OC and contribute to potential targeted therapies. In conclusion, our WGCNA identified candidate biomarkers for OC. Meanwhile, further studies were needed to make clear the underlying molecular mechanisms.

## CONFLICT OF INTEREST

All authors declare that they have no conflict of interest.

## AUTHOR CONTRIBUTIONS

M.W and L.Z conceived and performed the study. K.W and W.W designed the experiments. M.W and K.W wrote the manuscript. All authors read and approved the final manuscript.

## Supporting information

Table S1

Table S2

## Data Availability

The data that support the findings of this study are openly available in the MERAV (MERAV, http://merav.wi.mit.edu) database under the accession numbers GSM38087, GSM231870, GSM152673, GSM277697, GSM277737, GSM277738, GSM301703, GSM102478, GSM102483, GSM88956, GSM102426, GSM46830, GSM53029, GSM53054, GSM152581, GSM203626, GSM88974, GSM102445, GSM46831, GSM53036, GSM152577, GSM88973 and GSM76600.
