# Supplementary material for: Systematic prediction of key genes for ovarian cancer by co‐expression network analysis
Source: J Cell Mol Med. 2020 Apr 21;24(11):6298–307. doi: 10.1111/jcmm.15271 (PMC7294139; doi:10.1111/jcmm.15271)
Supplement: Supplementary file 1 — Table S1 [file JCMM-24-6298-s001.docx]

| **Table S1. Primer pairs for RT-PCR. Base sequences for determining β-ACTIN, COL1A1, BECN1 and ATG5 mRNA expression** | | |
| --- | --- | --- |
| **Oligonucleotide** | **Upstream Sequence** | **Downstream Sequence** |
| β-ACTIN | GGGACCTGACTGACTACCTC | TCATACTCCTGCTTGCTGAT |
| COL1A1 | AGACGAAGACATCCCACCA | GTCGCAGACGCAGATCC |
| BECN1 | GAGCGATGGTAGTTCTGGA | CCCGATGCTCTTCACCT |
| ATG5 | GCTTCGAGATGTGTGGTTT | GTTCTGCTTCCCTTTCAGTT |
