# Supplementary material for: Systematic prediction of key genes for ovarian cancer by co‐expression network analysis
Source: J Cell Mol Med. 2020 Apr 21;24(11):6298–307. doi: 10.1111/jcmm.15271 (PMC7294139; doi:10.1111/jcmm.15271)
Supplement: Supplementary file 2 — Table S2 [file JCMM-24-6298-s002.docx]

| **Table S2. Total genes of the blue module** | | | | | | | | | | |
| --- | --- | --- | --- | --- | --- | --- | --- | --- | --- | --- |
| **Genes** | | | | | | | | | | |
| UPK3B | EPYC | LOXL1 | SMURF2 | APCDD1 | IL1RAP | MS4A7 | SERPINH1 | TTC37 | FRMD6 | CTHRC1 |
| PANK1 | ECM1 | LOC403323 | SMYD3 | CILP2 | FAS | BCHE | FGF18 | KIAA0040 | OLFML2B | PCSK9 |
| LOC100130536 | EFNA3 | CUEDC1 | BMP3 | SAMD13 | IL7R | RARRES2 | SPHK1 | DOCK4 | SCARA3 | RLIM |
| COL1A1 | CELSR2 | LUM | ZBTB8A | LINC01341 | IL13RA1 | RECQL | P4HA2 | RASSF2 | BBS10 | SCG2 |
| TP53TG3HP | PLPP4 | TM4SF1 | SLC6A12 | PDIK1L | IL15 | BCL2L1 | GLB1L2 | SEC24D | COL6A3 | COL1A2 |
| RRN3P3 | ABCA1 | MEOX1 | BMP7 | LRRC71 | INHBA | RGS10 | CLIC3 | PLPPR4 | TMEM97 | LRRC32 |
| LOC100132323 | ARL13B | MEST | SLIT3 | CTSB | ITGA3 | RNASE1 | MED30 | KLHL21 | RHOF | LOC100130452 |
| CDH6 | SLC51A | MFGE8 | SNAI2 | CTSK | ITGAV | FKBP10 | CARMIL3 | GFPT2 | VANGL1 | WNT10A |
| ARPC1B | EML1 | MMP2 | CYP4F12 | CCDC80 | ANOS1 | RRAS | ACVR1 | COL12A1 | LRIG3 | COL5A2 |
| ARL4C | EMP1 | MMP11 | SOX9 | GLIPR2 | SWI5 | S100A1 | DEPDC7 | KCTD18 | MXRA5 | ALPK2 |
| SPRY1 | ENPEP | MMP13 | SPP1 | CTSZ | C2CD4B | SORT1 | OSMR | LYPD6B | PDE1A | ZDHHC20 |
| SEMA3A | EPHB3 | MMP14 | SSR3 | CYP1B1 | C2orf82 | S100A4 | XPR1 | PQLC3 | ZNF329 | KLHL5 |
| SPON2 | ETV4 | MN1 | ZFP36L2 | CYP11B2 | VGLL3 | S100A13 | GORAB | RNF212 | CLU | PAX8 |
| SPON1 | ETV5 | RPL7AP9 | HNF1B | DAPK1 | LGALS1 | CLEC11A | STK17B | GPR34 | KLK5 | LOC100130633 |
| CLGN | FAH | MSN | TCF15 | SPRED1 | LHX1 | CCL11 | LEMD1 | NKAIN3 | TNFRSF12A | EGFL6 |
| UNC13B | FAP | MT1M | TDO2 | DCN | LIMK2 | CXCL12 | SLIT2 | TRIM59 | NEIL1 | PLAU |
| NCOA2 | FBLN1 | MT2A | TEAD4 | SGMS2 | METTL10 | PIEZO2 | ITGBL1 | CYP2W1 | COL5A1 | RUBCNL |
| NPC2 | UNC5B | MT3 | TGFB1I1 | COL22A1 | SERTM1 | SH2D4A | ARHGAP29 | MCUB | SNORA71B | SPRY4 |
| ST6GALNAC2 | FAT2 | MTM1 | TGFB2 | OLFML2A | LMO2 | SFRP2 | SYTL4 | WDYHV1 | PLRG1 | FBXO32 |
| POSTN | FBLN2 | GADD45B | TGFB3 | DNM1 | C3orf80 | TUBBP5 | HOMER3 | RCBTB1 | COLEC12 | EPHX4 |
| CLDN16 | OTUD1 | ATF1 | TGFBI | DSC2 | LOX | NXN | CXCL14 | SLC10A3 | LBH | PCDH7 |
| PLK2 | PRR15 | NBL1 | TGM1 | COMP | RGCC | MCOLN3 | FAM57B | FZD1 | RPL39L | ZNF185 |
| NMU | FAM185A | NINJ2 | THBS2 | LRRC15 | GSN | 11-Sep | TMTC1 | HIST1H2AK | NGEF | CNN3 |
| RAB31 | KCTD20 | NNMT | THBS3 | COL6A6 | GUCY1A3 | CACNA2D3 | EVA1A | CRISPLD2 | COPZ2 | CLIC4 |
| DNAJB4 | FHL1 | NOS2 | THY1 | CD109 | GUCY1B3 | PRKCI | CMAHP | CMTM3 | ZBED2 | RAB23 |
| ESM1 | CD93 | NOV | KLF10 | CPE | SNX8 | SUSD2 | DYRK2 | HSPA12A | COL4A1 | UXS1 |
| CHI3L1 | KIFAP3 | NPR1 | TIMP3 | CRIP1 | LRP12 | TRPV5 | MEGF10 | TAF9B | GEM | FAM20A |
| CHKA | FLNA | NRAS | SERPING1 | CRIP2 | NRBF2 | KLK7 | CUL4B | ARMC5 | CLIC5 | FILIP1 |
| KLK8 | SULF1 | NT5E | TMOD1 | DOCK11 | A2M | KLK6 | SLC12A8 | COL11A1 | MFAP5 | C1QTNF5 |
| FZD10 | FMOD | ROR2 | TNFAIP6 | SELENOM | SH3KBP1 | ACKR3 | TPST2 | DOK7 | PRRX1 | FUT8 |
| CHML | FN1 | GPX8 | TPM1 | ISM1 | MNX1 | CEMIP | TUBB6 | ASPN | OSR2 | NTM |
| CHN1 | PPP1R13B | OMD | TWIST1 | CRYAB | FOXA1 | RCN3 | PLXDC2 | ST6GALNAC5 | MAP7D2 | WNT7A |
| PRKCDBP | TNS2 | TNFRSF11B | LOC729345 | ASB12 | TNC | SERTAD4-AS1 | MIR22HG | ACOT4 | PCOLCE | JSRP1 |
| AHNAK2 | DAAM2 | PAEP | S100A11P1 | SLC38A6 | ENPP7 | PTN | TMEM128 | TMEM158 | BHLHE41 | ZNF521 |
| CHRNA1 | MMD | NOX4 | LOC731484 | HAPLN3 | MYLK4 | WDFY4 | NKD2 | PDGFRL | COL3A1 | GPRC5B |
| XKR4 | FPR1 | COL5A3 | VCAM1 | VCAN | HNRNPCL1 | PVALB | DEGS1 | SUGCT | GAS1 | ATF7IP2 |
| C1QTNF3 | SH3BP4 | SERPINE1 | VEGFC | CSRP1 | IGFBP6 | PLEKHB1 | VAMP4 | COL8A2 | PLEC | PTRF |
